# Low level HIV viremia is more frequent under protease-inhibitor containing firstline therapy than under NNRTI-regimens

**DOI:** 10.7448/IAS.17.4.19828

**Published:** 2014-11-02

**Authors:** Frank Wiesmann, Patrick Braun, Mechthild Knickmann, Heribert Knechten

**Affiliations:** 1 HIV & Hepatitis Research, PZB Aachen, Aachen, Germany; 2 INSTO GmbH, INSTO, Aachen, Germany

## Abstract

**Introduction:**

An association of persistent low level viremia (LLV) below 500 copies/mL and a higher risk of therapy failure is still point of controversial discussion. Furthermore, it seems that LLV occurs more frequently in patients with protease-inhibitor regimens than in NNRTI- / or integrase-inhibitor containing therapies. The focus of this work was to assess the prevalence of LLV (50–200 copies/mL) and weak viremia (201–500 copies/mL) in firstline-treated patients according to their therapy regimen.

**Methods:**

A total of 832 and 944 patients from 23 German centres were under firstline therapy in 2012 and 2013, respectively. All patients received their therapy for more than 24 weeks. VL data was related to clinical data retrospectively including ART-composition, subdivided into NNRTIs (Efavirenz, Nevirapine), PIs (Atazanavir, Darunavir, Lopinavir) and INIs (Raltegravir). Low viremic patients were classified into two arms of 50–200 copies/mL (group A) and 201–500 copies/mL (group B).

**Results:**

Success of therapy was defined as <50 copies/mL and was observed in 90.0% and 91.1% (2012/2013), respectively. An additional 2.0% and 2.3% had LLV. The amount of viremic patients with VLs <500 copies/mL differed significantly between NNRTI-based firstline regimens 1.7% and 2.5% and PI-based regimens 4.8% and 5.7% (2012/2013), respectively. LLV was clearly less often observed in EFV-based- (1.6% and 1.1% [group A] / 0.4% and 0.4% [group B]) or NVP-based firstline therapies (1.0% and 3.6% [group A] + 0% and 0% [group B]) than in ATV-based- (7.5% and 3.8% [group A] + 1.5% and 2.5% [group B]), DRV-based- (2.9% and 3.0% [group A] + 2.2% and 0% [group B]) or LPV-based firstline therapies (1.6% and 3.3% [group A] + 0.8% and 2.5% [group B]) and also in parts for RAL-based regimens (0% and 3.7% [group A] + 0% and 1.9% [group B]).

**Conclusions:**

LLV is more often observed under PI-based firstline than under NNRTI-regimens. Only one NNRTI-patient of group B remained on therapy. A possible explanation for this discrepancy might be the fact that physicians seem to tolerate LLV more often in PI-regimens than in NNRTI-regimens due to a higher genetic barrier against resistance and it remains a point of discussion if constant LLV does affect immune recovery and risk of therapy failure.

